# USE OF A HOME SYMPTOM MANAGEMENT ZONE TOOL IN GERIATRIC PATIENTS UNDERGOING CANCER TREATMENT

**DOI:** 10.1093/geroni/igz038.3355

**Published:** 2019-11-08

**Authors:** Mary F Baker, Debra Shockey

**Affiliations:** 1 Virginia Commonwealth University School of Nursing, Richmond, Virginia, United States; 2 Virginia Commonwealth University, Richmond, Virginia, United States

## Abstract

Adult oncology practices are caring for a growing number of older adults with cancer. Evidence suggests that patients with cancer have higher emergency department (ED) utilization, particularly in patients 65 years of age and older and in those whose symptoms are poorly controlled. The ED is not the most appropriate nor most cost-effective option for addressing urgent care needs of patients undergoing cancer treatment. This quality improvement project is being conducted in a community medical oncology practice during usual cancer treatment education. Older adult patients starting or changing cancer treatment are presented with a zone tool (green-yellow-red) to inform decision-making at home regarding level of severity of current symptoms and recommended actions (e.g., you are stable, you need to call the office, you need to go immediately to the ED). The tool has been distributed to 28 patients with a goal of at least 30 by 9/20/19. Outcome measures include the number of ED visits in the intervention group compared to a pre-intervention sample of older adults from the same medical oncology office. Other outcome measures are acceptability of zone tool among clinical staff and patients. Preliminary data suggest suggest acceptability among staff and patients; and ease of use among clinical staff. Tracking of visits to the ED in the sample population and surveys of patients and staff are ongoing through October 2019.

